# Impact of Ventilation Discontinuation During Cardiopulmonary Bypass: A Prospective Observational Study

**DOI:** 10.3390/jcm14228215

**Published:** 2025-11-19

**Authors:** Tatyana Li, Azhar Zhailauova, Iwan Wachruschew, Aidyn Kuanyshbek, Shaimurat Tulegenov, Perizat Bukirova, Bekaidar Zhakupbekov, Ilya Nikitin, Dauren Ayaganov, Timur Kapyshev, Robertas Samalavicius, Andrey L. Melnikov, Theodoros Aslanidis

**Affiliations:** 1 Department of Anesthesia and Intensive Care, Heart Center CF “University Medical Center”, Astana 010000, Kazakhstan; leeanestrean@gmail.com (T.L.); dake281294@icloud.com (D.A.); 2 Department of Surgery, Nazarbayev University School of Medicine, Astana 010000, Kazakhstan; 3 Clinic of Emergency Medicine, Vilnius University, LT-03101 Vilnius, Lithuania; robertas.samalavicius@santa.lt; 4 Department of Anesthesiology and Intensive Care, Vestre Viken Hospital Trust, Ringerike Hospital, 3511 Hønefoss, Norway; 5 Intensive Care Unit & Anesthesia Department, Agios Pavlos General, Hospital of Thessaloniki, 55135 Thessaloniki, Greece; thaslan@hotmail.com

**Keywords:** mechanical ventilation, cardiopulmonary bypass CPB, cardiac surgery

## Abstract

**Background**: Discontinuing mechanical ventilation during cardiopulmonary bypass (CPB) is common but may adversely affect postoperative pulmonary function. This study aimed to evaluate the impact of stopping ventilation during CPB on postoperative gas exchange, radiographic findings, intensive care unit (ICU) length of stay (LOS), mortality, reintubation, re-exploration, and bleeding. **Methods**: A prospective observational study was performed involving adult patients scheduled for elective cardiac surgery requiring CPB. Participants were divided into ventilated and non-ventilated groups according to intraoperative strategy. Postoperative arterial carbon dioxide levels (PaCO_2_), arterial partial pressure of oxygen (PaO_2_), the PaO_2_/FiO_2_ ratio (P/F ratio), arterial oxygen saturation (SaO_2_), and the ratio of PaCO_2_ to minute ventilation (PaCO_2_/MV) were measured before the induction of anesthesia (within 5 min after transportation into the operating room), postoperatively within 5–10 min after transportation to the ICU, and in a 24 h postoperative period. Chest X-ray data, mechanical ventilation time, LOS in ICU, re-exploration, reintubation, and bleeding parameters were documented. Analyses were also conducted with the estimation of the age effect and BMI. **Results**: Individuals in the non-ventilated group exhibited lower postoperative P/F ratios and elevated postoperative PaCO_2_ and PaCO_2_/MV ratios. The difference in gas exchange leveled off within 24 h. There was no difference in the incidence of atelectasis (postoperatively in a 24 h period), mechanical ventilation time, LOS in ICU, or mortality. However, the incidence of bleeding was higher in the non-ventilated group (χ^2^ = 5.78, *p* = 0.016). Interestingly, postoperative PaCO_2_ and PaCO_2_/MV peaked in the 50-year age group. **Conclusions**: Continued mechanical ventilation during CPB correlates with better postoperative gas exchange, better CO_2_ clearance, and fewer bleeding events. The results suggest that maintaining low tidal volume ventilation during CPB may provide benefits, especially for patients aged 50 years.

## 1. Introduction

Despite widespread use of CPB, the optimal intraoperative ventilation strategy remains debated. CPB significantly reduces pulmonary perfusion leading to pulmonary tissue ischemia. When perfusion is restored, subsequent reperfusion injury accompanied by the release of free radicals, endothelial damage, and activation of the inflammatory cascade takes place [1,2,3]. These mechanisms already disrupt effective pulmonary gas exchange. Patients under general anesthesia are prone to the formation of lung atelectasis due to the influence of neuromuscular blocking agents, relaxed and shifted upward diagram, and supine position [4]. When ventilation discontinues during CPB, the effective gas exchange diminishes and alveoli are more prone to collapse resulting in an increased mean alveolar–arterial (A-a) gradient, intrapulmonary shunts [1], and greater extent of inflammation in lung parenchyma [5].

Some randomized controlled trials (RCTs) have investigated whether maintaining ventilation during CPB brings clinical benefits. The largest trial in this field, the MECANO trial, revealed no significant differences in overall postoperative complications between ventilated and non-ventilated groups [6]. However, for patients undergoing CABG, continued ventilation showed lower incidence of early respiratory failure and a need for prolonged ventilation or reintubation [7].

A study conducted by Davoudi et al. compared two groups of patients with continued or ceased ventilation during CPB and revealed that oxygenation status immediately postoperatively was better, and postoperative parameters of lung functionality, such as forced expiratory volume and forced vital capacity, were better in the ventilated group [8]. Moreover, the ventilated group demonstrated an earlier postoperative extubation time, leading the authors to conclude that protective continued ventilation during CPB yields superior pulmonary outcome improvements without increasing intraoperative risks. Other studies have highlighted potential secondary benefits such as decreased atelectasis incidence [9] and less fluid accumulation in extravascular pulmonary spaces in patients with maintained ventilation [10]. Several animal studies demonstrated that continued ventilation preserves the physiological structure of lung parenchyma, leading to more effective gas exchange and less inflammation during histopathologic lung analysis [5,11].

A recent retrospective study included 101 non-ventilated and 398 ventilated patients during CPB [12]. There were no reported discrepancies in the rate of postoperative pulmonary complications such as extended mechanical ventilation time, greater pleural effusion, and higher incidence of pneumonia between the groups [12].

Different hospitals apply varying ventilation protocols during cardiac surgeries involving extracorporeal circuits [13]. The differences between studies may be because of the absence of a universal ventilation protocol and the high inter-institutional variability in surgical practices. The heterogeneity in ventilation protocols may include variations in the ventilation mode (PCV, VCV), tidal volume, inspiratory pressure, respiratory rate, PEEP, FiO_2_, application of recruitment maneuvers, and timing of ventilation cessation. In addition, attention should focus both on short- and long-term postoperative pulmonary outcomes. A recent study performed by Rogers et al. demonstrated that preserving ventilation during mitral and aortic valve surgeries resulted in an improved lung function test at the 6–8-week follow-up and a better 6 min performance test at discharge [14]. Building on this evidence, the current study examines immediate postoperative gas exchange outcomes under different ventilation strategies.

The current investigation provides an assessment of the immediate and short-term physiological effects of discontinuing ventilation (MV) during CPB at three time points (before induction, after surgery, and in a 24 h period) that include gas exchange and ventilatory efficiency parameters such the PaCO_2_/MV ratio, as a marker of CO_2_ elimination efficiency. The findings of this study expand current clinical understanding of the effect of stopping ventilation during CPB and allow for better guidance of personalized ventilation strategies during cardiac surgery.

In this context, the present study aimed to compare the effects of continued versus discontinued mechanical ventilation during CPB, focusing on blood gas parameters in the immediate and 24 h postoperative period, X-ray findings, and other clinical data such as length of stay (LOS) in ICU, reintubation, mechanical ventilation time, incidence of re-exploration, mortality, and bleeding. We hypothesized that continuous ventilation during CPB improves early postoperative gas exchange compared with discontinuation.

## 2. Materials and Methods

### 2.1. Study Design

This prospective, single-center observational study was conducted at the UMC Heart Center (Astana, Kazakhstan). The study protocol received approval from the institutional ethics committee (Approval Number: 2022/01-137/CI dated 20 October 2022), and written informed consent was obtained from all participants prior to enrollment in accordance with the principles outlined in the Declaration of Helsinki.

### 2.2. Patient Population

The study included adult patients scheduled for elective cardiac surgery requiring cardiopulmonary bypass (CPB) between 1 January 2023 and 1 June 2024. Patients were eligible for inclusion if they met the following criteria:

- Age ≥ 18 years;
- Elective coronary artery bypass grafting (CABG) and/or valve surgery;
- Intraoperative requirement for CPB.

Patients were excluded based on the following criteria:

- Reoperation;
- Presence of severe chronic pulmonary disease (GOLD stage III–IV), to avoid confounding due to pre-existing pulmonary impairment;
- Preoperative mechanical ventilation or the presence of a tracheostomy;
- Refusal to provide informed consent.

Participants were divided into two groups according to the intraoperative ventilation strategy employed during CPB (both groups were comparable by ASA):

- The ventilated group included 59 patients who received low tidal volume mechanical ventilation throughout CPB.
- The non-ventilated group included 64 patients in whom mechanical ventilation was temporarily discontinued upon initiation of CPB, as determined by a surgeon in a case-by-case scenario depending on the complexity of a procedure, when low tidal volume ventilation interfered with certain surgical steps.

### 2.3. Anesthesia, Intraoperative Ventilation, and Postoperative Ventilation Management

All patients underwent general anesthesia with induction achieved using propofol, fentanyl, and rocuronium. Anesthesia was maintained with sevoflurane and a continuous propofol infusion of 4–6 mg/kg/h according to the department’s protocol.

In the non-ventilated group, mechanical ventilation was stopped after CPB initiation for the period required by the surgeon to implement certain surgical interventions. The stop ventilation time lasted from 1 to 187 min. In the ventilated group, a lung-protective ventilation strategy according to the department’s protocol was maintained during CPB in PCV/VCV mode with the following settings: tidal volume, 2–3 mL/kg ideal body weight; FiO_2_, 0.5; respiratory rate, 6–8 breaths/min; positive end-expiratory pressure (PEEP), +5 cm H_2_O, which was maintained consistently during surgery. CPB was conducted under standardized conditions using non-pulsatile flow (2.4 L/min/m^2^), spontaneous normothermia, and alpha-stat pH management. In the ICU, patients were supported with ventilation in BiPAP mode with the following settings: tidal volume, 6–8 mL/kg ideal body weight; FiO_2_, 0.3–0.5; respiratory rate, 12–14 breaths/min; positive end-expiratory pressure (PEEP), 5–10 cm H_2_O. Sedation depth was documented as a Richmond Agitation-Sedation Scale (RASS) score from −3 to −5. Weaning protocol was based on the internal hospital protocol adopted from the principles of the ICU Liberation Bundle (A–F, Society of Critical Care Medicine).

### 2.4. Data Collection

Arterial blood gas (ABG) samples were collected at three time points according to the department’s protocol: before induction of anesthesia, (FiO_2_, 0.21, within 5 min after transportation into OR), postoperatively within 5–10 min after transportation to the ICU, and in a 24 h postoperative period. Arterial blood analysis was carried out using the same analyzer, and operators were blinded to the groups. The following gas exchange parameters were measured: arterial partial pressure of carbon dioxide (PaCO_2_, mmHg), arterial partial pressure of oxygen (PaO_2_, mmHg), PaO_2_/FiO_2_ ratio (P/F ratio), and arterial oxygen saturation (SaO_2_). Additional perioperative variables were recorded, including duration of CPB, aortic cross-clamp time; mechanical ventilation (MV) time; stop ventilation time; X-ray results immediately after surgery in the ICU (5–10 min, before extubation) and in a 24 h postoperative period; days in ICU; and baseline demographics. X-ray images were assessed by two blinded physicians with an inter–rater agreement (κ) equal to f1.0.

### 2.5. Study Outcomes

Primary and secondary outcomes were pre-specified before data collection. The primary outcome of the study was the comparison of the P/F (PaO_2_/FiO_2_) ratio between the ventilated and non-ventilated groups. Secondary outcomes included the assessment of PaCO_2_, PaO_2_, the PaCO_2_/MV (PaCO_2_/minute ventilation, mmHg/L/min) index, X-ray findings, ICU stay, mortality, reintubation, re-exploration, and bleeding. The PaCO_2_/MV index is a surrogate for ventilatory efficiency and dynamically assesses how effectively the lungs eliminate CO_2_ per unit of ventilation. A sample size assessment was performed assuming a moderate effect size (Cohen’s d = 0.4) for the difference in the primary outcome parameters, a two-sided α = 0.05, and power = 0.80.

### 2.6. Statistical Analysis

Statistical analysis was performed using the open-access R environment (R 4.4.2). Normality was tested with the Shapiro–Wilk test, and continuous variables are expressed as mean ± standard deviation (SD) or median with interquartile range [IQR], depending on the distribution. Categorical data are presented as absolute numbers and percentages. The Student’s *t*-test or the Mann–Whitney U test was used for continuous variables. The Kruskal–Wallis test was applied to continuous variables comparing more than two groups. Pearson and Spearman correlations were also calculated. A *p*-value of <0.05 was considered statistically significant.

## 3. Results

**Figure 1:**
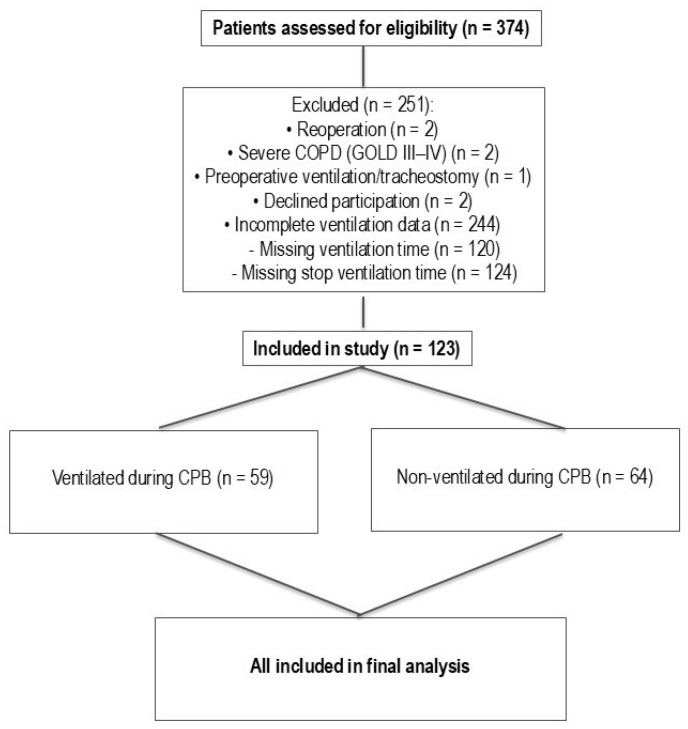
Strobe diagram of patient screening and selection.

Figure 1 represents patient screening and subsequent selection. Table 1 shows the demographic data for the ventilated and non-ventilated groups. Age, height, weight, and BMI were consistent between groups. There were 21 females and 43 males (*n* = 64) in the non-ventilated group and 18 females and 41 males (*n* = 59) in the ventilated group. A statistically significant difference in gender distribution was not observed between groups (chi-square, *p* = 0.94).

Following detailed data analysis, the two groups of cardiac surgery patients were compared during the perioperative period. Careful comparison revealed statistically significant differences in gas exchange immediately after surgery in patients admitted within 5–10 min to the ICU. All patients in the ICU were supported with BiPAP ventilatory support with identical ventilation parameters: tidal volume, 6–8 mL/kg ideal body weight; FiO_2_, 0.3–0.5; respiratory rate, 12–14 breaths/min; and positive end-expiratory pressure (PEEP), 5–10 cm H_2_O. The non-ventilation group exhibited higher arterial PaCO_2_ levels in the ICU (mean 44.38 mmHg) compared with the ventilated group (mean 40.56 mmHg; *p* = 0.026). Similarly, postoperative PaO_2_ values were lower in the non-ventilated group (mean 127.8 mmHg) than in the ventilated group (mean 144.3 mmHg; *p* = 0.044). The P/F ratio, a marker of oxygenation efficiency, was reduced by 14% in the non-ventilated group (291.7) compared with the ventilated group (339.2; *p* = 0.028). Statistical analysis was performed applying Welch’s *t*-test. Table 2 represents the means of statistically different parameters between groups.

To account for potential confounding effects on the primary outcome, an inverse probability of treatment weighting (IPTW) analysis based on a logistic propensity score was conducted. The model included age, BMI, ejection fraction, CPB time, cross-clamp time, pulmonary artery systolic pressure (PASP), and surgery type. The IPTW analysis indicated that ventilation during CPB was associated with a higher mean P/F ratio in the ICU (IPTW RoM 1.214, 95% CI 1.085–1.357, *p* = 0.001; stabilized IPTW RoM 1.203, 95% CI 1.034–1.400, *p* = 0.017). Likewise, a stabilized IPTW model with covariate adjustment yielded a comparable estimate (RoM 1.231, 95% CI 1.065–1.424, *p* = 0.005), which states that ventilation during CPB is linked to a higher mean of P/F ratio in the ICU (by 23%) after accounting for measured confounders.

However, at the 24 h postoperative mark, these differences were largely resolved. PaCO_2_ levels remained slightly higher in the non-ventilated group (*p* = 0.054), but PaO_2_, SaO_2_, and P/F ratios were comparable between the groups (all *p* > 0.1). Postoperative gas exchange parameters analyzed in the ICU after 24 h were collected after extubation according to the department’s protocol and are represented in Table 3.

**Figure 2:**
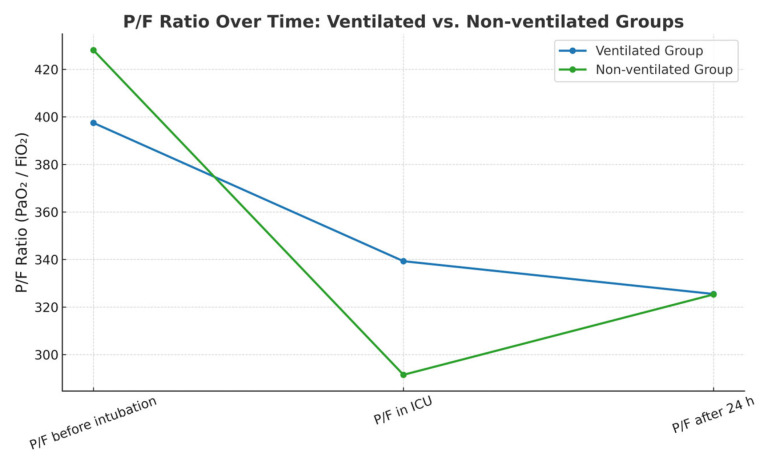
P/F ratio at three time points. There is a significant drop in the P/F ratio by the end of the surgery (measured in the ICU within 5–10 min after transportation, on BiPAP mode of ventilation) in each group (398 → 339, *p* < 0.001 for the ventilated group and 428 → 292, *p* < 0.001 for the non-ventilated group). In the ventilated group, the P/F ratio values in the ICU and after the 24 h postoperative mark are very close (339 → 325, *p* = 0.42), reflecting almost no change in oxygenation. Conversely, in the non-ventilated group, oxygenation improved after 24 h compared with the P/F ratio values in the ICU (292 → 326, *p* = 0.045).

Further comparisons of the P/F ratio at three perioperative time points showed statistically significant changes of the parameter within each group. Perioperative time points were registered per the department’s protocol in perioperative patient management. At the initiation of anesthesia, both groups exhibited similar P/F ratio values. However, immediately following surgery, the group with continued ventilation demonstrated significantly higher oxygenation, as evidenced by a statistically significant difference in P/F ratios. After 24 h, the P/F values converged again, showing no significant difference between groups. Table 4 and Figure 2 below illustrate the distribution of P/F values across the three perioperative time points for both the ventilated and non-ventilated groups.

Minute ventilation (MV) values were comparable between the ventilated and non-ventilated groups at induction and by the completion of the surgery (5.71 vs. 5.75, 6.17 vs. 6.04). Minute ventilation at the end of the surgery was registered in the ICU within 5 min after transportation from the surgery unit. In the ventilated group, MV values were higher at surgery completion, indicating the need for more ventilatory support (6.17 vs. 5.71, *p* = 0.012). The situation was similar in the non-ventilated group, although the increase in postoperative ventilatory support was borderline significant (5.75 vs. 6.04, *p* = 0.057). Table 5 demonstrates the abovementioned parameters.

Next, the PaCO_2_/MV index, used as an indicator of ventilatory efficiency, showed no significant difference before CPB in non-ventilated and ventilated groups, respectively (7.95 vs. 7.13; *p* = 0.44), but was slightly elevated after CPB in the non-ventilated group (7.44 vs. 6.79; *p* = 0.059), suggesting the possible presence of sample size limitation.

**Figure 3:**
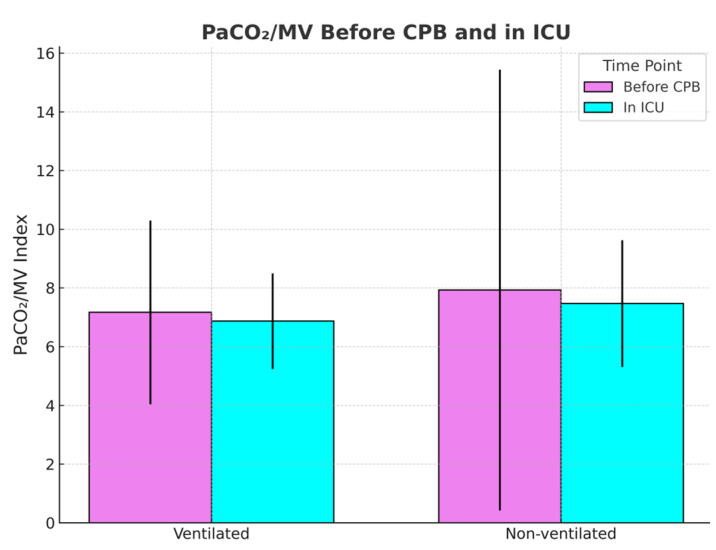
PCO_2_/MV index before and after CPB between the ventilated and non-ventilated groups.

**Figure 4:**
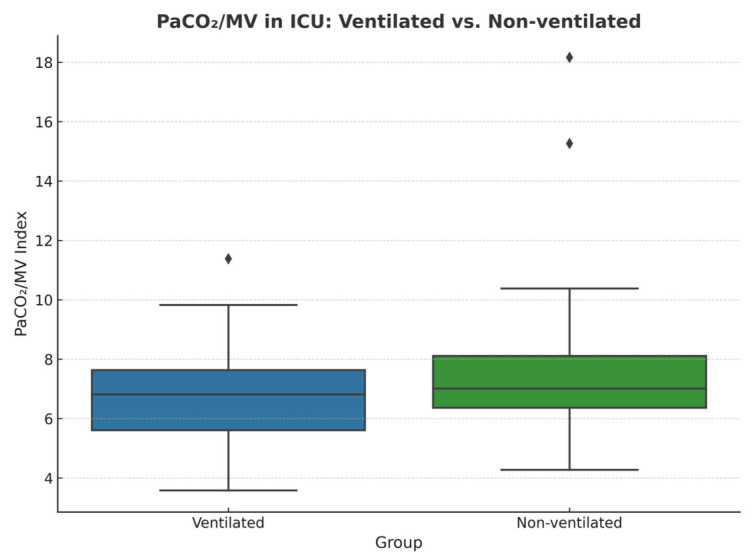
Comparison of PaCO_2_/MV in ICU between ventilated and non-ventilated groups, measured within 5–10 min after transportation to the ICU from a surgery unit. Outliers are demonstrated as diamonds.

The obtained *p*-value may suggest that statistical significance could be achieved with a larger sample size. Figure 3 and Figure 4 compare the PaCO_2_/MV index before and after CPB and the PaCO_2_/MV index in the ICU after the surgery for each group, respectively.

**Figure 5:**
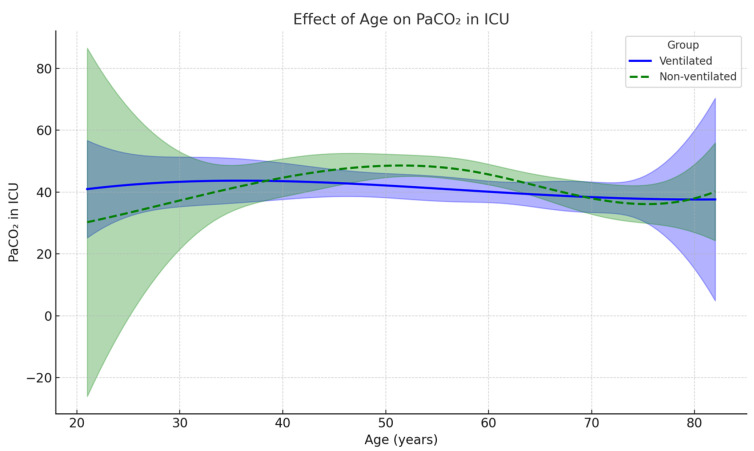
A linear regression model using restricted cubic splines demonstrates PaCO_2_ values in ICU across age groups between ventilated and non-ventilated groups. The group with discontinued ventilation demonstrates decreased CO_2_ removal that peaked at the age of 50 years, with the subsequent normalization in the ventilation efficiency toward the extremes of age. PaCO_2_ values with 95% CI at selected ages demonstrate a non-linear pattern with wide variability, particularly in the non-ventilated group.

Before comparing the age effect of the PaCO_2_/MV index, the effect of age was observed on PaCO_2_. A linear regression model with restricted cubic splines (df = 4) was applied to derive the potential nonlinear effect of age on PaCO_2_ levels between ventilated and non-ventilated groups. Shaded areas indicate 95% confidence intervals around the predicted means. As a result, the model indicated a moderate fit (R^2^ = ~0.15), although none of the spline terms for age were significant (df = 4, *p* = 0.83, *p* = 0.15, *p* = 0.98, *p* = 0.73). Although the distribution of PaCO_2_ is curved toward the middle ages in the non-ventilated group, the effect of ventilation was also not significant between the groups (*p* = 0.72). Figure 5 demonstrates these findings. Considering the PaCO_2_/MV index across age groups, PaCO_2_/MV showed considerable overlap between the non-ventilated and ventilated groups, presenting similar patterns as PaCO_2_ distribution across age.

Because the effect of ventilation did not introduce variability in the age–PaCO_2_ relationships, we combined the data to explore the correlation pattern between age and PCO_2_/MV. The combined data showed a U-shaped or curved relationship with PCO_2_/MV after surgery, with higher values observed in middle-aged patients and lower values towards extremes.

**Figure 6:**
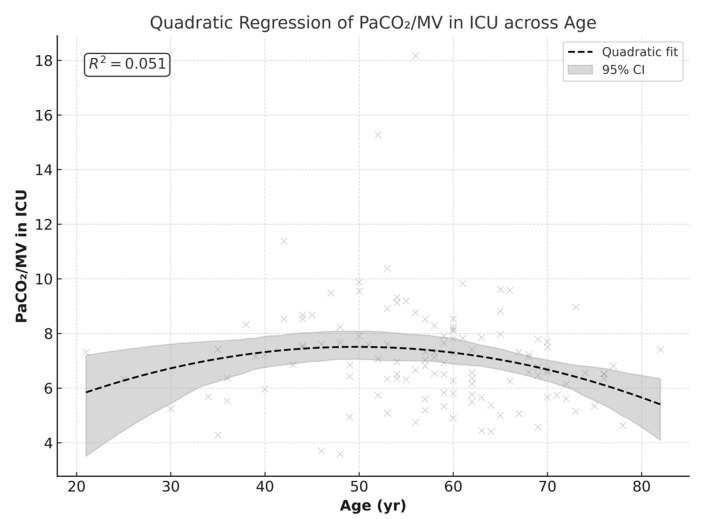
PaCO_2_/MV index in ICU (postoperatively) distributed by age: between ventilated and non-ventilated groups combined. Gray “×” marks represent individual patient data.

A quadratic regression model showed a linear correlation between age and PCO_2_/MV by 50 years of age (≈49.7 years), with a predicted peak maximum value of 7.5 (β = +0.20, *p* = 0.046). At older ages, the slope became negative, creating a curved decline (β = −0.002, *p* = 0.028). Although the linear terms of the curved correlation trend are statistically significant, the overall fitness of the model explains only 5% of the variation in PCO_2_/MV (R^2^ ≈ 0.051), representing a weak correlation. Figure 6 demonstrates this quadratic regression.

**Figure 7:**
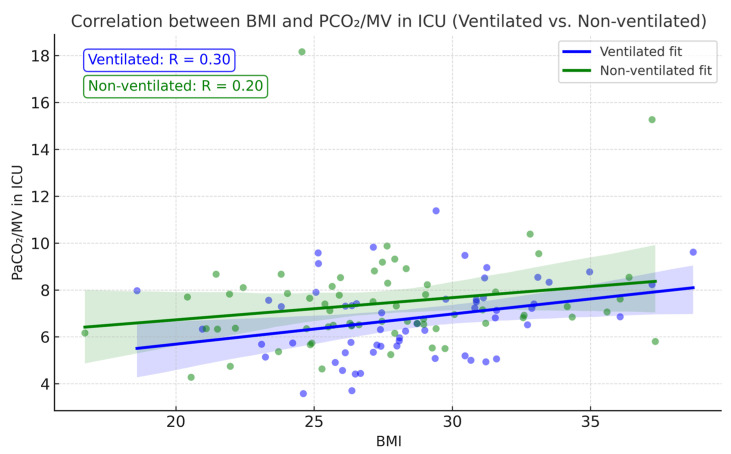
Correlation between BMI and PCO_2_/MV across ventilated and non-ventilated groups.

Postoperative PaCO_2_/MV also showed a slight correlation with patient BMI: the higher the BMI, the higher the index (Figure 7), correlating with theoretical expectations of an increase in atelectasis formation with greater weight. Correlation analysis between BMI and PCO_2_/MV showed a positive correlation coefficient of r = 0.296, *p* = 0.023 in the ventilation group. Although the correlation coefficient remained positive (r = 0.203; *p* = 0.11) in the non-ventilated group, it was not statistically significant.

Post-extubation chest X-rays revealed no statistically significant difference in atelectasis incidence: eight cases in the non-ventilated group vs. seven cases in the ventilated group. Atelectasis was defined on X-rays as collapsed lung tissue in the form of linear shadows of increased density. At 24 h, three new cases of atelectasis were detected in the non-ventilated group, while the ventilated group had none, although this difference was not statistically significant. Table 6 represents the X-ray findings at both postoperative periods. X-ray findings in the ICU (postoperatively, during 5–10 min) and at a 24 h postoperative mark were captured according to the department’s protocol of postoperative patient management.

The median value of the stop ventilation time observed in the non-ventilated group was 45.5 min, with the IQR in the range of 29.75–60.0 min. Correlation analyses showed that the stop ventilation time in the non-ventilated group was moderately associated with longer cross-clamp and CPB times (r ≈ 0.47 and 0.41, respectively).

**Figure 8:**
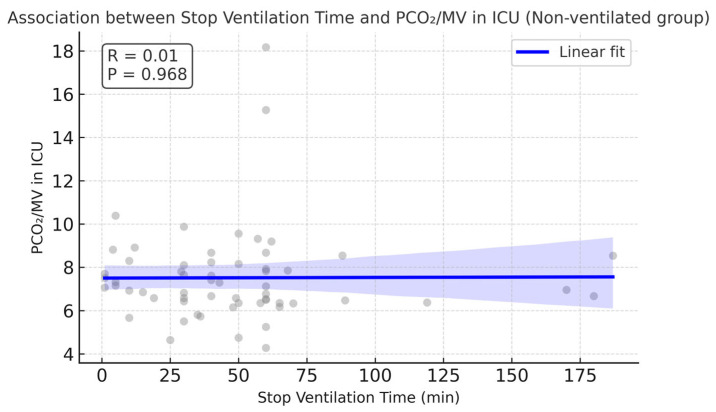
No correlation observed between PaCO_2_/MV in the ICU and stop ventilation time in the non-ventilated group. Pearson correlation, r = 0.005, *p* = 0.97. The regression line is almost flat, supporting the absence of an association.

Interestingly, CPB time in the non-ventilated group was higher compared with the ventilated group (108.89 vs. 90.05 min, *p* = 0.033). No correlation was found between stop ventilation time and LOS in the ICU, incidence of postoperative pulmonary complications, or PaCO_2_/MV in the ICU. Figure 8 demonstrates absence of correlation between PaCO_2_/MV in the ICU and stop ventilation time in the non-ventilated group. There were no significant differences in LOS in the ICU (*p* = 0.35). Single episodes of lethal outcome were observed in each group (1 case out of 59 in the ventilated group and 1 case out 64 in the non-ventilated group). Table 7 demonstrates the values of the compared parameters.

**Figure 9:**
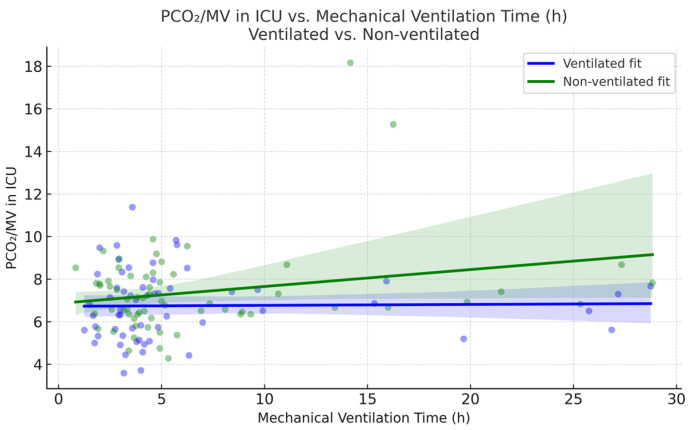
Mechanical ventilation time and PaCO_2_/MV in ICU association across ventilation modes.

Mechanical ventilation time was registered from arrival in the ICU until extubation. Mechanical ventilation time was comparable between the ventilated and non-ventilated groups (6.25 vs. 6.75, *p* = 0.68). In the non-ventilated group, mechanical ventilation time showed a weak and possibly borderline significant association with PaCO_2_/MV after surgery (Pearson r = 0.24, *p* = 0.061). No such association was noted for the ventilated group (Figure 9).

**Figure 10:**
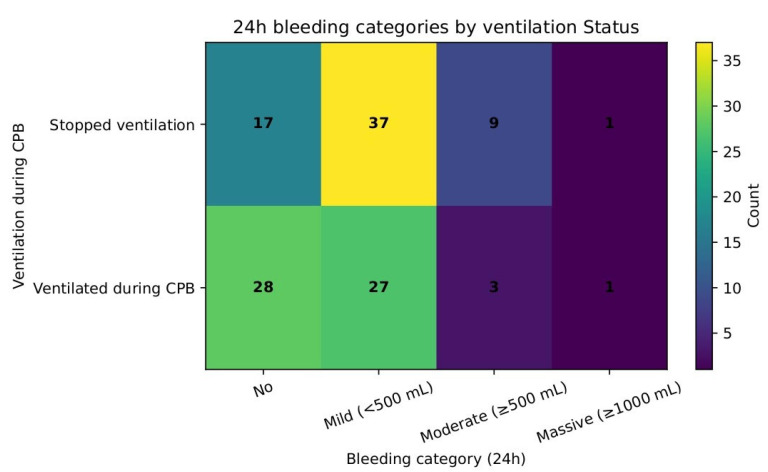
Bleeding severity in 24 h between ventilated and non-ventilated groups.

Bleeding severity was compared in a 24 h assessment from the draining chest tubes and stratified as none, mild, moderate, and massive (Table 8). A chi-square test showed that there was no difference in bleeding severity between the groups (χ^2^ = 7.06, df = 3, *p* = 0.07). Figure 10 shows these distributions.

**Figure 11:**
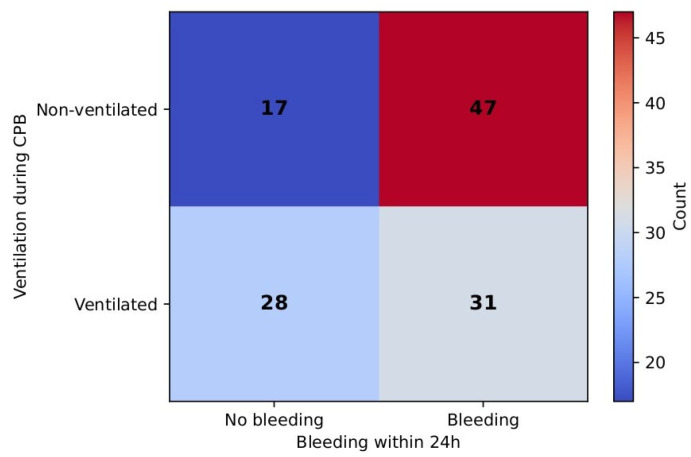
Bleeding between groups.

When binarization was performed as no bleeding (0 mL) vs. any bleeding (≥0 mL), a significantly lower incidence of bleeding was observed in the ventilated group. In the non-ventilated group, 73.4% of patients experienced bleeding, compared to 52.5% in the ventilated group. The chi-square test result was significant (χ^2^ = 5.78, *p* = 0.016) and was supported by Fisher’s exact test (*p* = 0.024). The odds of bleeding were 2.5 times higher in the non-ventilated group (OR = 2.5). RR comprised 0.72 (95% CI: 0.54–0.95), which suggests a 28% reduction in any bleeding being ventilated. Figure 11 demonstrates this correlation.

**Figure 12:**
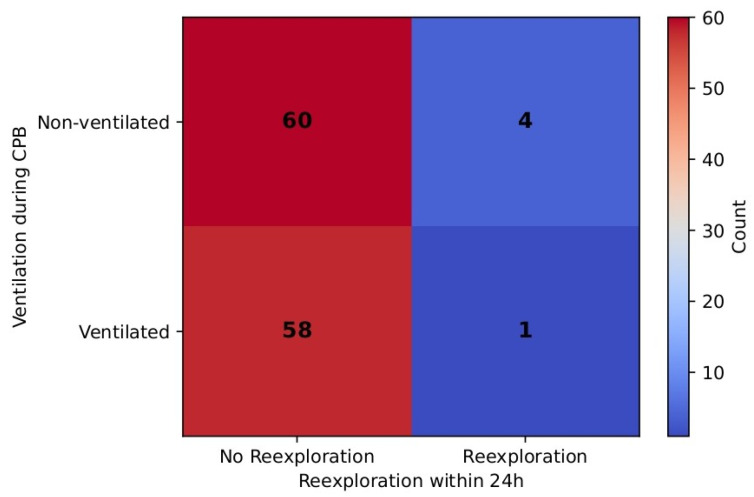
Re-exploration difference between the groups.

Of note, re-exploration incidence within 24 h was a rare event in the ventilated group (1.7%) as well as in the non-ventilated group (6.7%), although the difference was not statistically significant (Fisher’s exact *p* = 0.37). RR (0.27, 95% CI [0.031, 2.358]) favored the ventilated group, but CI is wide, thus suggesting uncertainty due to the small number of events. Table 9 and Figure 12 demonstrate these results.

## 4. Discussion

The current study evaluatesthe effects of intraoperative ventilation discontinuation during CPB on postoperative gas exchange and lung function. The findings demonstrate that temporary cessation of mechanical ventilation was associated with a transient but statistically significant increase in PaCO_2_ and a reduction in the extent of oxygenation during the immediate postoperative period. This suggests that the interruption of ventilation may reduce CO_2_ elimination and disturb efficient oxygenation in the early recovery phase. Importantly, these gaseous discrepancies were resolved in a 24 h postoperative period, indicating a transient nature of the effect. Multiple regression analysis shows that considering the confounding effect of age, BMI, ejection fraction, CPB time, cross-clamp time, PASP, and surgery type, the mean P/F ratio in the ICU in the ventilated group stays higher, supporting the beneficial effect of continued ventilation during CPB.

The observations coincide with previous studies demonstrating that better ventilation and oxygenation status were observed in patients with continued low tidal volume ventilation during the immediate postoperative period. Davoudi et al., in a randomized controlled study with 50 patients in each group, demonstrated better post-CPB PaO_2_ values in patients who remained on low tidal volume ventilation mode compared with patients who had ceased ventilation [8]. In addition, the ventilated group showed better postoperative forced expiratory volume as well as forced vital capacity results [8]. Similar animal experiments demonstrated that low-frequency ventilation retained during CPB resulted in better PaO_2_ and less histological lung damage compared with subjects where ventilation was not provided or only PEEP of +5 cm H_2_O was supported [5].

A previously conducted meta-analysis of randomized controlled trials applied the evidence of better oxygenation status in patients on continuous ventilation during the immediate postoperative period, although a beneficial reduction in pulmonary complications was not observed [15]. The largest RCT, the MECANO trial, demonstrated that stop ventilation strategy was not inferior to continued ventilation in terms of mechanical ventilation time, mortality, postoperative respiratory failure, and incidence of reintubation [6]. Considering other pulmonary complications, a recent study concluded that cessation of ventilation during CPB does not reduce the incidence of infection during the postoperative recovery period [16]. However, in patients undergoing mitral and aortic valve surgeries, preserved ventilation was associated with better forced vital capacity and an increase in forced expiratory volume during 1 s by 0.19 and 0.135 L, respectively, in the postoperative period. Moreover, the ratio of forced expiratory volume during 1 s over forced vital capacity was 5% higher in the 6–8-week postoperative follow-up for the group with preserved ventilation [14]. Moreover, this study demonstrated that the soluble receptor for advanced glycation end products (sRage) was significantly higher in the preserved ventilation group, although the ventilation mode was low frequency rather than low tidal volume ventilation. In addition, the 6 min walk test performed upon discharge showed better performance in the group with continued ventilation [14]. This may suggest that continuing ventilation during CPB may result in better postoperative lung function parameters that could influence long-term patient recovery.

Ventilation management was standardized at induction and resulted in almost identical values between the ventilated and non-ventilated groups before CPB (5.71 vs. 5.75, *p* = 0.84). In turn, postoperative minute ventilation values measured in the ICU demonstrated a slight increase in minute ventilation demand in both ventilated and non-ventilated groups (6.17 vs. 6.04 L/min, *p* = 0.43). From a ventilatory support perspective, cessation of ventilation during CPB did not require more ventilatory support; although the increase in postoperative ventilatory support was statistically significant in the ventilated group (from 5.71 ± 1.33 to 6.17 ± 1.00 L/min, *p* = 0.012), the statistical difference was borderline in the non-ventilated group (from 5.75 ± 1.05 to 6.04 ± 0.77 L/min, *p* = 0.057), suggesting the need for a larger patient sample. The results may also suggest that changes in lung mechanics triggered by anesthesia and CPB may require greater postoperative ventilatory support.

The linear regression model demonstrates that age has a nonlinear effect on postoperative PaCO_2_ levels measured in the ICU. The model shows the nonlinear relationship toward distribution of PaCO_2_, which peaked in the 50-year-old range in the non-ventilated group. Considering that the minute ventilation values were comparable between groups, the PaCO_2_/MV parameter was used as a measure of ventilatory efficiency regarding CO_2_ clearance. In the non-ventilated group, the higher PaCO_2_/MV index suggested a trend toward less efficient CO_2_ elimination, although it did not reach statistical significance (*p* = 0.059). Quadratic regression analysis revealed an age-dependent U-shaped relationship, with PaCO_2_/MV reaching a peak at around 50 years. This indicates that middle-aged patients may be more susceptible to impaired ventilatory efficiency postoperatively, independent of ventilation strategy. The U-shaped correlation supports the findings of a previous study that assessed atelectasis formation in 243 patients by comparing CT atelectasis before and after anesthesia induction [4]. The study revealed that after anesthesia induction, atelectasis formation increased up to 50 years of age and then declined with older age [4]. As people age, lung elasticity decreases, thus increasing the incidence of atelectasis formation, which may explain the trend of increased PaCO_2_/MV up to 50 years. However, with further increase in age, the trend of atelectasis formation decreases. A possible explanation may be the closure of small airways. With age, small airways are more prone to closure during preoxygenation at supine position, thus trapping nitrogen. Trapped nitrogen in turn may reduce the incidence of atelectasis formation in closed airways [4]. Similarly, higher BMI correlated positively with PaCO_2_/MV in ventilated patients, supporting the hypothesis that obesity predisposes patients to reduced lung compliance and greater risk of postoperative atelectasis. In contrast, no significant association was observed in non-ventilated patients.

Radiographic evidence of atelectasis was not significantly different between groups, yet new cases detected only in the non-ventilated group suggest that subtle effects on lung recruitment may not be fully captured by chest X-ray alone. However, a study conducted on 60 randomly distributed patients showed that low tidal volume ventilation (3 mL/kg, respiratory rate (RR) 6/min, and PEEP +5 cm H_2_O) demonstrated a lower incidence of postoperative atelectasis of 10%, compared with 36.6% (*p* < 0.05) in the group in which only PEEP of +5 cm H_2_O (CPAP mode of ventilation) was preserved during CPB [9]. On the other hand, X-ray may underestimate atelectasis incidence, and ultrasound could provide a more sensitive bedside evaluation of postoperative atelectasis [17] in the areas where air does not interfere with the trajectory of an ultrasound beam. In the current results, while radiographic atelectasis was slightly more frequent in the non-ventilated group at the 24 h postoperative mark, this did not reach statistical significance. While decreased or absent lung ventilation and a relaxed diaphragm could contribute to atelectasis formation during anesthesia, the CT results of one study showed that a 32% increase in postoperative heart mass led to the increased incidence of atelectasis formed in the subjacent lung area in patients after CABG [18]. Thus, the effect of increased cardiac muscle should be considered in future studies.

Stop ventilation time correlated moderately with cross-clamp and CPB durations but showed no association with ICU stay, PaCO_2_/MV, or other pulmonary complications. Mechanical ventilation time demonstrated only a weak and borderline association with PaCO_2_/MV in the non-ventilated group, suggesting limited predictive value. Interestingly, we also observed higher re-exploration rates in the non-ventilated group, although incidences were rare. The assessment of real effect may require a greater sample size. In addition, the 24 h assessment point shows that higher incidences of bleeding were observed in the non-ventilated group (χ^2^ = 5.78, *p* = 0.016), although the severity of bleeding was comparable (χ^2^ = 7.06, df = 3, *p* = 0.07) between the groups. No incidence of intraoperative transfusions in either group was observed. In contrast, Rodriguez-Blanco et al. reported higher incidences of coagulopathy and mediastinal re-exploration in the group with preserved lung ventilation and perfusion via a shunt to the pulmonary artery [19]. Stop ventilation time had a moderate positive correlation with CPB time (r ≈ 0.41). This could be explained by several factors. The longer exposure to extracorporeal circuits induces a stronger inflammatory response as well as a higher extent of coagulopathic derangements [20]. The prolonged CPB time elongates the ischemic state of lung tissues further contributing to the reperfusion–inflammation injury [3]. Prolonged CPB time is also linked to the complexity of surgical procedures. Thus, further investigations could be implemented in this direction.

Although the observed physiological changes were transient and did not impact the ICU stay or need for reintubation, they may be clinically relevant in vulnerable populations, such as in patients with pre-existing lung disease, obesity, or advanced age. During CPB, pulmonary circulation is significantly decreased or avoided [21]. Longer CPB times may exacerbate ischemia–reperfusion lung injury, further disturbing delicate lung parenchyma and subsequently ventilation and oxygenation status in postoperative recovery [3]. An animal study showed that pigs on preserved lung perfusion and ventilation had higher P/F ratios (90 min after CPB start: 244 ± 57 vs. 126 ± 64 mmHg, *p* < 0.001), fewer polymorphonuclear cells during pulmonary histopathologic analysis, and less thickening of alveoli septa, which is vital for gas exchange, compared with standard CPB without preservation of lung perfusion and ventilation [11]. Thus, the results of the study suggest that ischemia–reperfusion injury could be mitigated by maintaining minimal ventilation during CPB. Another human observational study demonstrated that ventilation preservation during CPB resulted in reduced extravascular pulmonary fluid (530 vs. 672, *p*= 0.028) and faster extubation time (3.6 vs. 4.8 h) in cardiac surgery patients undergoing CABG [10], highlighting further benefits of continued lung ventilation.

Overall, the findings support the physiological rationale for continued ventilation during CPB to reduce immediate postoperative derangements in gas exchange. Meanwhile, the transient nature of these differences raises questions regarding the clinical significance of this practice in the long term. However, the study assessing long-term effects in patients after mitral and aortic valve surgeries demonstrates better lung functionality and 6 min walking test results at follow-up. In addition, the overexpression of sRage biomarkers in the ventilated group may play a role in ischemia–reperfusion injury caused by CPB and could inform clinical decisions concerning the optimal ventilation strategy during CPB [14].

Larger multicenter randomized trials are needed to determine whether maintaining ventilation during CPB translates into improved long-term outcomes in lung functional capacity and patient physical fitness. Furthermore, the application of sRage and its clinical implications are of particular interest. It would also be interesting to examine how lung functional capacity may change across age groups.

### 4.1. Strengths and Limitations

The strength of this study is the granular perioperative dataset with repeated gas exchange measurements (before surgery start, immediately post-op and at 24 h), allowing us to separate early from transient effects. We also examined complementary PaCO_2_/MV, age- and BMI-dependencies, and radiographic outcomes, providing a multidimensional view of pulmonary physiology after CPB. Additionally, the single-center design of the study enhances internal consistency.

Limitations include its single-center design and modest sample size, which may have contributed to the borderline *p*-values. For the primary outcome, with a target power of 80%, a two-sided test, α = 0.05, and an allocation ratio of 1:1, the sample size was underpowered, suggesting that each group should have gathered ≥99 participants. Management heterogeneity, including recruitment maneuvers after CPB or immediately after ICU arrival, and further ventilator setting adjustments were not documented and may have confounded immediate ICU and at 24 h gas exchange differences. We also did not document changes in extravascular liquid accumulation in the lungs, which could have been measured by ultrasound during the postoperative period. Finally, a chest X-ray assessment could have been complemented by an ultrasound-guided bedside assessment of lung atelectasis in the ICU immediately after surgery; however, the number of physicians performing such assessments was limited. It would be beneficial if objective lung imaging such as ultrasound or CT were included in the future studies to validate radiographic results.

### 4.2. Recommendations for Future Research

Larger multicenter randomized trials would be beneficial in assessing the immediate and long-term pulmonary characteristics after CPB between ventilated and non-ventilated groups. Correct assessment of extravascular liquid accumulation in the lungs and postoperative bleeding through fibrinogen level would diminish the confounding effects of reperfusion injury and postoperative bleeding. It would be also beneficial if objective lung imaging such ultrasound or CT were included in future studies to validate radiographic results.

## 5. Conclusions

Temporary cessation of ventilation during CPB transiently impairs gas exchange; maintaining low-tidal-volume ventilation may be beneficial, particularly in patients with limited pulmonary reserve. Although these changes are resolved within 24 h, the findings support the consideration of individualized ventilation strategies, particularly for patients with a compromised pulmonary reserve.

## Figures and Tables

**Table 1:** Demographic data between the ventilated and non-ventilated groups.

	Ventilated Group, Mean	Non-Ventilated Group, Mean	*p*-Value
Age (year)	56.8	57.4	0.77
Height (cm)	166.3	165.8	0.70
Weight (kg)	78.9	76.04	0.20
BMI (kg/m^2^)	28.6	27.5	0.17

**Table 2:** Immediate postoperative gas exchange values between ventilated and non-ventilated groups. Postoperative values were registered within 5–10 min of transportation to the ICU from a surgery unit.

Parameter	Ventilated, Mean, mmHg	Non-Ventilated, Mean, mmHg	95% CI Mean Difference	*p*-Value
P/F in ICU	339.18 ± 98.88	291.70 ± 135.50	[4.83, 90.13]	0.028
PaCO_2_ in ICU	40.56 ± 5.87	44.38 ± 12.03	[−7.23, −0.39]	0.026
PaO_2_ in ICU	147.35 ± 44.99	127.85 ± 60.43	[0.35, 38.65]	0.044

**Table 3:** Ventilation and oxygenation parameters at the 24 h postoperative mark.

Parameter at 24 h	Ventilated, Mean	Non-Ventilated, Mean	95% CI Mean Difference	*p*-Value
P/F, mmHg	325 ± 130.94	326 ± 81.41	[−40.67, 39.37]	0.974
PaO_2_, mmHg	105.7 ± 31.54	97.8 ± 24.42	[−3.82, 16.78]	0.134
PaCO_2_, mmHg	42.1 ± 3.78	44.0 ± 6.58	[−3.88, 0.11]	0.054

**Table 4:** P/F ratio at three perioperative time points.

Parameter	Ventilated, Mean	Non-Ventilated, Mean	95% CI Mean Difference	*p*-Value
P/F before intubation	398 ± 52.88	428 ± 184.24	[−77.99, 17.80]	0.215
P/F in ICU	339.18 ± 98.88	291.70 ± 135.50	[4.83, 90.13]	0.028
P/F at 24 h	325 ± 130.94	326 ± 81.41	[−40.67, 39.37]	0.974

**Table 5:** Minute ventilation values at induction of anesthesia and postoperatively between ventilated and non-ventilated groups.

Minute Ventilation, Hours	Ventilated, Mean, L/min, (95% CI)	Non-Ventilated, Mean, L/min, (95% CI)	*p*-Value
Surgery Start	5.71 ± 1.33 [5.36 to 6.06]	5.75 ± 1.05 [5.49 to 6.01]	0.84
ICU	6.17 ± 1.00 [5.91 to 6.43]	6.04 ± 0.77 [5.85 to 6.23]	0.43

**Table 6:** ICU X-ray after extubation and in a 24 h period.

	Ventilated Group	Non-Ventilated Group
Normal	52	56
Atelectasis	7	8
In 24 h		
Atelectasis	0	3
Normal	59	61

**Table 7:** Other parameters are compared between the non-ventilated group and ventilated group.

Parameter	Ventilated, Mean	Non-Ventilated, MEAN	95% CI Mean Difference	*p*-Value
Cross-clamp time (min)	56.98 ± 35.95	56.84 ± 34.11	[−12.83, 13.11]	0.98
CPB time (min)	90.05 ± 37.51	108.89 ± 56.67	[−36.60, −1.08]	0.033
Mechanical ventilation time (hours)	6.25 ± 6.68	6.74 ± 6.30	[−2.83, 1.84]	0.67
LOS in ICU	2.80 ± 3.46	2.33 ± 1.13	[−0.44, 1.38]	0.345

**Table 8:** Bleeding estimation at 24 h period: massive > 1000 mL; moderate > 500 mL; mild < 500 mL.

	Ventilated Group	Non-Ventilated Group
Massive: >1000 mL	1	1
Moderate: >500 mL	3	9
Mild: <500 mL	27	37
None	28	17

**Table 9:** Re-exploration incidence in 24 h.

	Ventilated Group	Non-Ventilated Group
No	58	60
Yes	1	4

## Data Availability

The original contributions presented in this study are included in the article. Further inquiries can be directed to the corresponding author.
